# P-947. The Impact of Physician-Driven Antimicrobial Stewardship Intervention with Education, Post-Prescription Review and Feedback on Empirical Antimicrobials Initiated in the Medical Intensive Care Unit

**DOI:** 10.1093/ofid/ofaf695.1150

**Published:** 2026-01-11

**Authors:** Jeffry Samuel Selvakumar, Jerlin Prasanna Victor Selvaraj, Keerthana Karthikeyan

**Affiliations:** Sugam Hospital, Chennai, Tamil Nadu, India; Sugam hospital, Chennai, Tamil Nadu, India; Sugam hospital, Chennai, Tamil Nadu, India

## Abstract

**Background:**

Alarming increase in inappropriate antimicrobial usage has led to antimicrobial resistance, a global threat. Developing countries like India, which lack qualified infectious disease specialists and robust antimicrobial stewardship programs (ASPs) in most hospitals, are particularly vulnerable to this issue. We assessed the effectiveness of ASP driven by a physician with internal medicine background and training in infectious disease.

**Methods:**

This prospective, single-centre cohort study, conducted over a 10 month period at a tertiary care hospital, included patients aged ≥ 18 years receiving study antimicrobials for 48 hours or more in medical intensive care unit (ICU). This consisted of 3 phases: a baseline observation phase of 4 months where antimicrobials initiated in ICU were evaluated for appropriateness without provision of any recommendations; an education and training phase for 2 months with workshops and seminars; and an intervention phase for 4 months where post-prescription review with feedback and recommendations were provided sequentially by a physician from 48 hours after the initiation of empirical antimicrobial therapy in ICU until discharge. The primary outcome of the study was days of therapy (DOT) of the study antimicrobials per 1000 study patient days (PD).

**Results:**

Overall, 105 patients were recruited in the baseline phase and 110 patients were recruited in the intervention phase. DOT/ 1000 PD decreased from 2258.9 in the baseline phase to 1672 in the intervention phase (*P* < 0.001). Inappropriate antimicrobial use reduced from 61 % to 38.2% between the intervention and baseline phase (*P* = 0.14). Unnecessary double coverage and inappropriate choice of empirical antibiotics reduced from 54.3 % and 38.1 % in baseline phase to 26.4% and 10.9 % in the intervention phase (*P* = 0.007 and *P* < 0.001, respectively). The median number of antibiotics used per patient decreased from 3 (1,5) to 2 (1,4) between the baseline and the intervention phase (*P* < 0.001).The de-escalation rate based on culture susceptibility increased in the intervention phase versus the baseline phase (78.2 % vs 37.8 %; *P* < 0.001).

**Conclusion:**

The physician-driven ASP will be an effective strategy in reducing consumption of antimicrobials in a lower-middle-income country, India.

**Disclosures:**

All Authors: No reported disclosures

